# Safety and feasibility during early implementation of robotic-assisted percutaneous coronary intervention

**DOI:** 10.3389/fcvm.2026.1731900

**Published:** 2026-02-25

**Authors:** Thuong Dung Ho, Manh Cuong Nguyen, Tran Tran Nguyen, Long Tran, Huy Nguyen, Dil Mai, Tuong Mien Ngo, Duc Chinh Nguyen, Chi Cuong Tran, Loc Vu, An Viet Tran, Khiem Ngo, Thach Nguyen, Aravinda Nanjundappa

**Affiliations:** 1 Vinmec Central Park International Hospital, Ho Chi Minh City, Vietnam; 2 Can Tho S.I.S General Hospital, Can Tho, Vietnam; 3 Can Tho University of Medicine and Pharmacy, Can Tho, Vietnam; 4 School of Medicine, Tan Tao University, Tay Ninh, Vietnam; 5 Cardiovascular Research Laboratories, Methodist Hospital, Merrillville, IN, United States; 6 Department of Medicine, University of Texas Rio Grande Valley at Valley Baptist Medical Center, Harlingen, TX, United States; 7 Cleveland Clinics, Cleveland, OH, United States

**Keywords:** coronary artery revascularization, coronary artery disease, PCI—percutaneous coronary intervention, robotic interventions, robotic-assisted percutaneous coronary intervention

## Abstract

**Background:**

Robotic-assisted Percutaneous Coronary Intervention (R-PCI) is an advanced technique offering potential advantages for both patients and interventional cardiology practices. In 2023, Can Tho S.I.S General Hospital implemented its first robotic system for PCI procedures. This study aimed to evaluate the safety and clinical outcomes during the early learning curve of R-PCI.

**Methods:**

This prospective study included all patients undergoing R-PCI with the CorPath GRX Vascular Robotic System at Can Tho S.I.S General Hospital from April to September 2023. Baseline patient characteristics, procedural details, and six-month follow-up data were collected. Primary outcomes included Clinical Success, defined as <30% residual diameter stenosis in the target vessel without major adverse cardiovascular events (MACE) during hospitalization (e.g., death, myocardial infarction, target-vessel revascularization, stroke), and robotic success, defined as achieving clinical success with partial manual support or no manual support.

**Results:**

Thirty-one patients (mean age 64.5 ± 10 years; 64.5% male) with 37 lesions underwent R-PCI. Robotic success was achieved in 94.6% (35/37 lesions), comprising 21 lesions (56.8%) performed as full R-PCI and 14 lesions (37.8%) requiring partial manual support (exclusively for IVUS catheter manipulation). Manual conversion occurred in 5.4% due to inadequate guiding-catheter back-up and slow-flow. No MACE occurred during hospitalization; however, one patient died from a stroke during the six-month follow-up.

**Conclusion:**

This study demonstrates high success rates and minimal complications for R-PCI in its early implementation phase. Nevertheless, the stroke-related death observed during follow-up highlights the need for long-term studies to comprehensively assess R-PCI's safety and efficacy.

## Introduction

The integration of robotics into medical procedures presents potential advantages for both patients and healthcare professionals, driving significant expansion in their adoption across various surgical fields (1). Of particular note in cardiovascular intervention is the emerging field of Robotic-assisted Percutaneous Coronary Intervention (R-PCI) (2–6). R-PCI offers distinct benefits over traditional manual PCI by enhancing procedural outcomes through steadier and more precise movements, minimizing operator radiation exposure, and improving physician ergonomics by eliminating the necessity of wearing a heavy lead apron (2–7).

The rationale for implementing R-PCI lies in its ability to reduce procedural variability and enhance stent deployment accuracy, addressing common limitations in traditional PCI. Robotic systems in other fields, such as hip arthroplasty, have shown enhanced accuracy in implant positioning, correlating with reduced long-term complications and improved patient outcomes (8). Although comprehensive long-term studies specific to R-PCI are needed, initial findings suggest potential parallels in improving cardiovascular outcomes through precision in robotic-assisted stent placement.

Safety benefits, particularly in terms of reduced radiation exposure, are one of the primary motivations for robotic-assisted surgery adoption. As operator experience with R-PCI increases, studies show a gradual decrease in fluoroscopy time, underscoring a learning curve in optimizing procedure duration without compromising patient safety (9). Notably, while R-PCI tends to require longer fluoroscopy time than manual PCI, the total contrast volume remains similar, suggesting efficient procedural control even in the robotic setting (10).

This study assesses the safety, feasibility, and clinical success of R-PCI during the early learning phase at Can Tho S.I.S General Hospital, with a focus on clinical and robotic success rates, procedural challenges, and complications. By addressing these factors, we aim to contribute to the limited data on R-PCI's feasibility in high-volume cardiac care settings, guiding future strategies for robotic system implementation in similar clinical environments.

## Methods

### Inclusion criteria

From April 2023 to September 2023, all patients diagnosed with coronary artery disease (CAD) and indicated for PCI, in accordance with clinical guidelines, were selected (11). The patients provided informed consent to undergo robot-assisted PCI.

### Exclusion criteria

**Figure 1:**
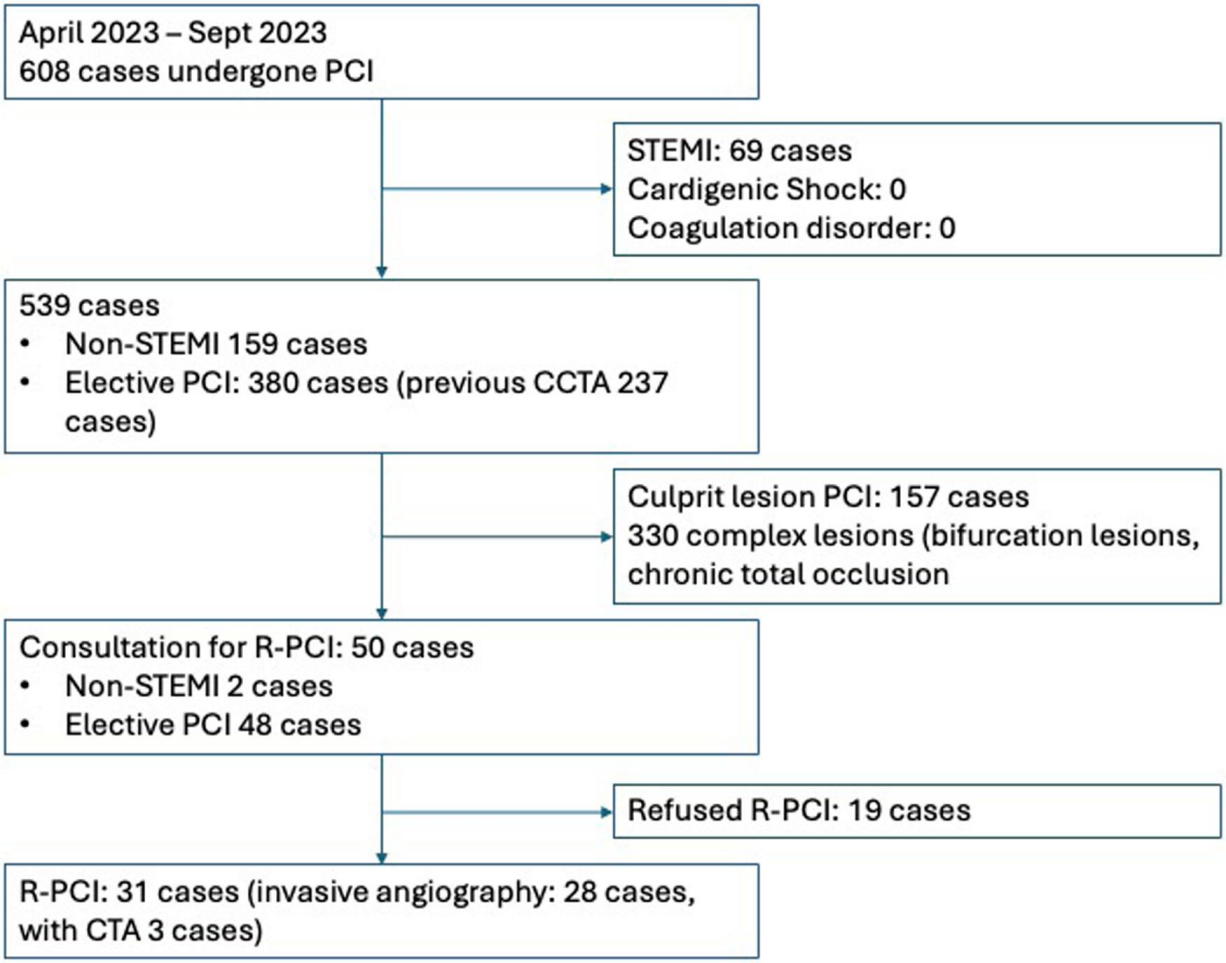
Study flow chart.

Patients were excluded from the study if they met any of the following criteria: (1) Patients with ST segment elevation myocardial infarction (STEMI), cardiogenic shock, sepsis, coagulation disorder; (2) contraindication to standard antiplatelet therapy (heparin, aspirin, clopidogrel, or other P2Y12 inhibitors); (3) complex PCI (bifurcation lesions), CTO (Chronic Total Occlusion). The study flow chart presented in Figure 1.

### Clinical data

Upon admission, demographic and clinical characteristics, including age, gender, body mass index (BMI), smoking status, and medical history (hypertension, atrial fibrillation, coronary artery disease, diabetes mellitus, dyslipidemia, prior cerebral stroke, and chronic kidney disease) were recorded. Additionally, laboratory measurements were conducted to assess troponin T, creatinine, and lipid profiles (total cholesterol, triglycerides, high-density lipoprotein-C, and low-density lipoprotein-C), along with echocardiographic evaluations.

### Robotic system

**Figure 2:**
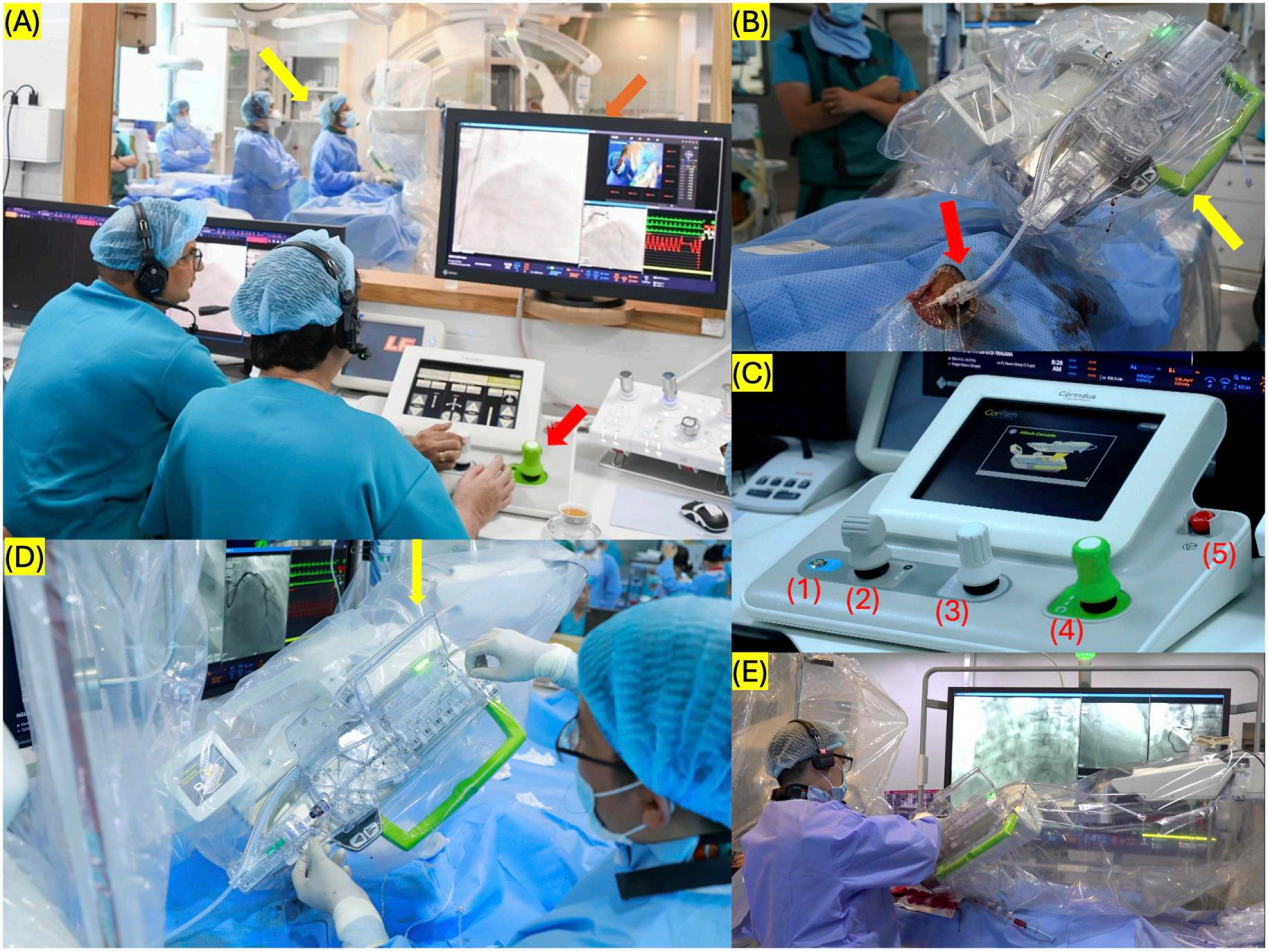
Codius robotic system for percutaneous coronary intervention (PCI) **(A)** Red arrow: An operator remotely controls the movement of PCI devices, sitting down outside cardiovascular intervention room; yellow arrow: an operator setup device stands inside cardiovascular intervention room; orange arrow: power vision monitor. **(B)** A single-use cassette connected with a vascular access sheath, red arrow: sheath, yellow arrow: cassette) **(C)** Control console: (1) Turbo Button, (2) Balloon/Stent Joystick, (3) Guidewire Joystick, (4) Guide Catheter Joystick, (5) Emergency Stop. **(D,E)** Operator setup guidewire on cassette. The photograph was taken during one of the center's initial interventional cases. The increased personnel count (including the proctoring specialist from the manufacturer and the standby team ready for manual conversion) was necessary for training purposes and to ensure maximum safety during the initial implementation phase.

The system CorPath GRX robotic (Corindus, Siemens Healthineers Company, Waltham, MA, USA) connected to digital subtraction angiography (DSA) is implemented for R-PCI (Figure 2). The system included the Corpath GRX control console, Corpath GRX Extrended reach arm and Corpath GRX robotic drive.

### Procedure protocol

The R-PCI team comprised two experienced interventional cardiologists with prior training in R-PCI. One interventional cardiologist operated the robot arm via the control panel, while the other assisted in the intervention room, managing the installation of instruments (guidewires, dilator balloons, and stents) into the robotic arm system. Additionally, a nurse assisted with the equipment, and a technician was responsible for adjusting the DSA machine and the robotic system.

Before the intervention procedure, patients received a full dose of antiplatelet drugs (aspirin, clopidogrel, or ticagrelor). Unfractionated heparin was administered intravenously at a dose of 70–100 units per kilogram of body weight before the insertion of the interventional guide (11).

Cardiac catheter access was obtained via the radial artery or femoral artery. The catheter's end was connected to a pressure-measuring wire before being attached to the drive gear system. The cassette system was connected to the Y-lock and then installed into the robotic arm. Finally, the system was positioned appropriately, and the procedure commenced.

### Study endpoints

The success of R-PCI implementation was evaluated based on two aspects: clinical and **robotic**. Clinical success was defined as the successful placement of the stent at the target lesion, with quantitative coronary angiography (QCA) showing less than 30% diameter stenosis, a TIMI flow grade of 3, and no major adverse cardiac events (MACE) during the hospital stay (12). **Robotic** success was defined as achieving clinical success while completing the intervention with either **Full R-**PCI or with **partial manual support**. **Full R-PCI** refers to the entire PCI procedure being performed exclusively from the robotic console with no manual manipulation of intracoronary devices. **Partial manual support** refers to necessary manual aid where the procedure is ultimately completed robotically. **Manual conversion** was defined as the necessity to entirely disengage the robotic system to complete the remaining PCI steps manually (2, 13).

Additionally, post R-PCI laboratory data included radiation time (minutes), patient radiation exposure (µGy·m²), procedure time (from the arterial puncture until the last interventional device was completely withdrawn from the guide catheter), the dose-area product for technical support personnel (µSv), and contrast volume (mL).

A six-month follow-up was conducted, including an assessment of target lesion failure. Furthermore, all major adverse cardiac events (MACEs) were meticulously recorded. These included cardiac death, target-vessel myocardial infarction (MI), clinically driven target lesion revascularization, any death, any MI, and any clinically driven revascularization.

### Institutional ethical approval

This study was approved by the Ethical Committee of the Can Tho University of Medicine and Pharmacy with the approval numb: 23.001.NCS/PCT-HDDD. Additionally, informed consent was obtained from each participant, and the research protocol adhered to the principles outlined in the Declaration of Helsinki.

### Statistical analysis

All clinical features, procedural features, and clinical outcome results are illustrated as counts and percentages for categorical variables and as mean ± standard deviation or median (interquartile range) for continuous variables.For categorical variables, comparisons between groups (e.g., gender, prevalence of risk factors, clinical success/failure rates) were performed using the Chi-square (chi^2^) test or Fisher's exact test when the expected frequencies were less than five. For continuous variables that exhibited a normal distribution, differences between the two independent groups were evaluated using the unpaired Student's *t*-test. For continuous variables that did not follow a normal distribution or for ordinal data, the Mann–Whitney *U*-test was used to compare the central tendency between the two independent groups. A two-sided *p*-value of <0.05 was considered statistically significant. Statistics were performed using Stata 15 (StataCorp. 2017. Stata Statistical Software: Release 15. College Station, TX: StataCorp LLC).

## Results

In total, 608 patients underwent PCI from April 2023 to September 2023, which included 69 cases with STEMI, 159 cases with non-STEMI and 380 cases of unstable angina. Based on the lesion characteristic, 50 patients were referred for R-PCI. Finally, 31 patients agreed for R-PCI.

### Demographics and clinical features

31 patients (64.5% male) meeting the predefined inclusion criteria underwent R-PCI. Of these patients, 7 exhibited a single vessle disease, 17 had two vessle disease, and 7 had three vessle disease. Overall, 37 coronary lesions were intervened with R-PCI. Baseline clinical characteristics of the cohort, including demographic and comorbidity profiles, are detailed in Table 1. Notably, due to the study's conduct within a specialized in stroke, a higher prevalence of prior cerebrovascular events was observed in comparison to similar studies.

**Table 1:** Baseline demographic and clinical characteristics.

Characteristics		Patient (*n* = 31)	Percentage (%)
General Information			
Age (years), mean ± SD		64.5 ± 10	
<60		11	35.5
60–69		9	29.0
70–80		10	32.3
>80		1	3.2
Gender	Male	20	64.5
	Female	11	35.5
Smoking (*n*, %)		16	51.6
Hypertension		31	100
Hyperlipidemia		27	87.1
Prior cerebral stroke		10	32.3
Prior myocardial infarction		11	35.5
Atrial fibrillation		1	3.2
Diabetes		11	35.5
Heart failure		0	0
Chronic kidney disease		3	9.7
Indication for percutaneous coronary intervention			
Stable angina		29	93.5
Acute coronary syndrome		2	6.5
Laboratory parameters			
Triglycerides (mmol/L)			2.1 ± 1.1
Total cholesterol (mmol/L)			4.5 ± 0.9
High-density lipoprotein-C (mmol/L)			1.0 ± 0.3
Low-density lipoprotein-C (mmol/L)			2.8 ± 0.7
Creatinine (μmol/L)			91.6 ± 20.2
EF (%)			66.8 ± 7.6

Among the 37 lesions treated, the left anterior descending (LAD) artery accounted for 23 cases, the right coronary artery (RCA) for 10 cases, while the left circumflex artery (LCX) and obtuse marginal (OM) artery accounted for three and one case, respectively. Based on the American College of Cardiology/American Heart Association (ACC/AHA) classification of coronary lesions, the majority were classified as type A (40.5%), as detailed in Table 2.

**Table 2:** Procedural characteristic.

Characteristics		Patient (*n* = 31)	Percentage (%)
Accessing sites	Right radial arterial	25	80.6
	Left radial arterial	0	0
	Right femoral artery	6	19.4
Stent/PCI	One	22	71.0
	Two	8	25.8
	Three	1	3.2
Vessel disease	1-vessel disease	7	22.6
	2-vessel disease	17	54.8
	3-vessel disease	7	22.6
Intervened Vessel	One	25	80.6
	Two	6	19.4
Intra-vascular ultrasound (IVUS)		14	37.8
Lesion characteristics (*n* = 37)			
Lesion location	Left anterior descending artery	23	62.2
	Left circumflex artery	3	8.1
	Right coronary artery	10	27.0
	Obtuse marginal artery	1	2.7
ACC/AHA lesions classification	Type A/B1	23	62.2
	Type B2/C	14	37.8
Lesion length	≤20 mm	8	21.6
	>20 mm	29	78.4
Stent diameter (mm)		3.2 ± 0.4	
Stent length (mm)		32.0 ± 9.3	
Quantitative Coronary Angiography	70–90%	27	73.0
	>90%	10	27.0
Calcification Degree	Severity	4	10.8
	Mild/moderate	14	37.8
	No calcium	19	51.4
Ballooning before stenting (yes)		31	83.8
Ballooning after stenting (yes)		28	75.7
Procedure time (min)		40.8 ± 18.2	
Radiation time (min)		17.3 ± 7.6	
Air kerma radiation dose for patient dose (µGym^2^)		1,019 (629.7–1,541)	
Patient's Radiation Exposure (µGym^2^)		9,569 ± 6,071	
Technical supporter's dose-area product (µSV)		0.25	
Contrast volume (mL)		7155 ± 45.8	

**Figure 3:**
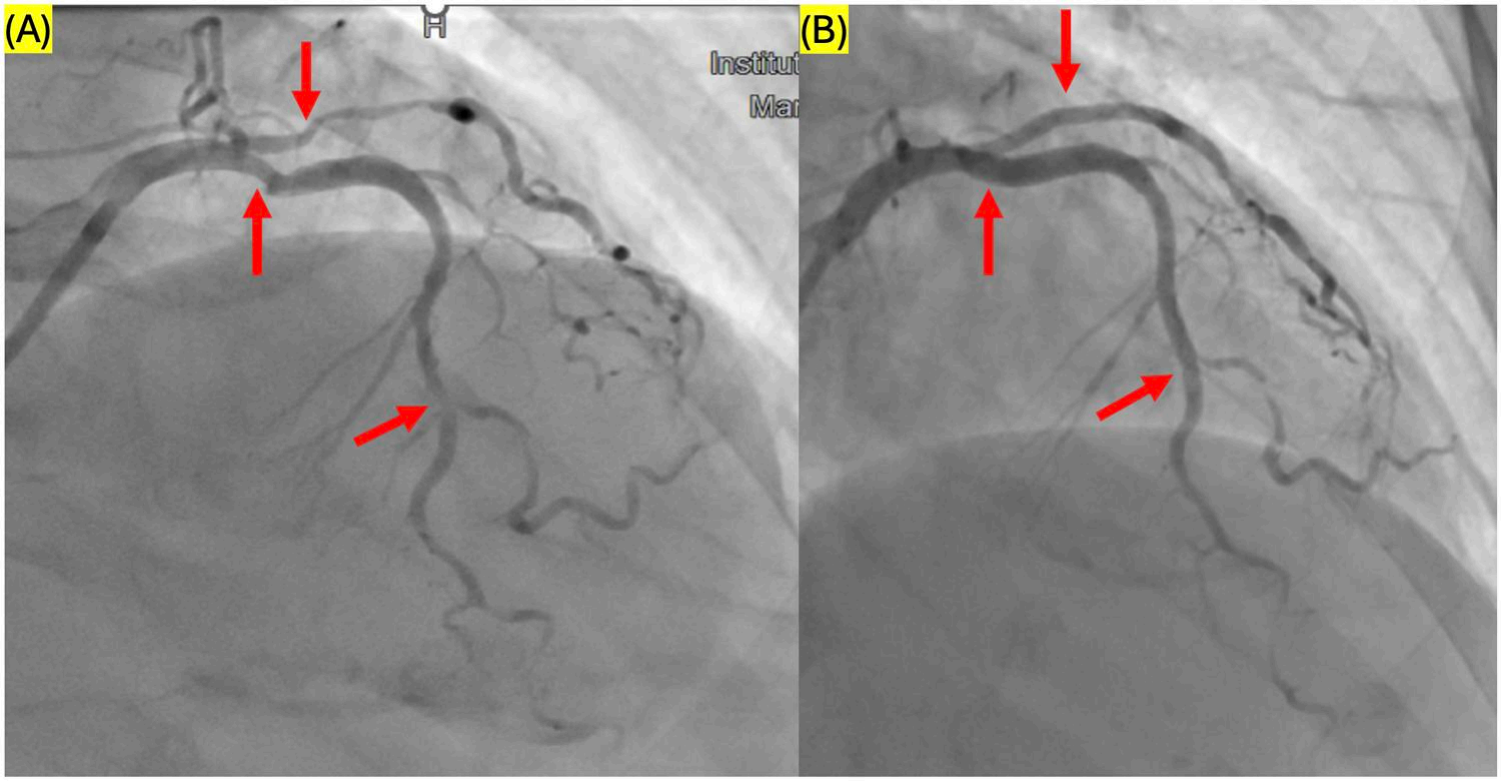
**(A)** Severe stenosis on left anterior descending artery (LAD) I, LAD III and obtuse marginal branch OM1, **(B)** stented with 1 drug-eluting stent (DES) 3.5 mm × 23 mm on LAD I and 01 stent 2.5 mm × 18 mm on LAD III, 01 stent 2.5 mm × 23 mm on OM1.

Eight patients received two stents, and one patient required the placement of three stents during a single procedure (Figure 3). Additionally, two cases necessitated conversion to manual intervention. The average procedure time was 40.8 ± 18.2 min, with a total fluoroscopy time of 17.3 ± 7.6 min. Patient radiation exposure was measured at 9,569 ± 6,071 µGy·m^2^, with a contrast volume of 155 ± 45.8 mL utilized.

### Procedural outcomes

The clinical success rate was 97.3%. Only 2.7% of lesions exhibited TIMI grade 2 flow, specifically observed in one case with acute side-branch occlusion. The **robotic success** rate was 94.6% (35/37 lesions). Among these, **full R-PCI** was achieved in 21 lesions, where the entire procedure was completed without any manual contact with intracoronary devices. **Partial manual support** was required in 14 lesions (37.8%); notably, this assistance was strictly limited to the manual advancement and withdrawal of the IVUS catheter due to current system incompatibility. No manual support was required for the manipulation of coronary wires, balloons, or stents in any of the successful cases. **Manual conversion** was required for 2 lesions (5.4%) due to inadequate guiding-catheter backup and slow-flow. A comprehensive summary is presented in Table 3.

**Table 3:** In-hospital outcomes.

Characteristics		Lesion (*n* = 37)	Percentage (%)
Clinical success		36	97.3
Robotic success	Full R-PCI	35	94.6
	Partial Manual Support^a^	14	37.8
	Manual conversion	2	5.4
Myocardial infarction post PCI		1	2,7
Stent thrombosis post PCI		0	0
Post PCI TIMI flow	TIMI I	0	0
	TIMI II	1	2.7
	TIMI III	36	97.3

^a^ IVUS only, Thrombolysis in Myocardial Infarction (TIMI), Percutaneous coronary intervention (PCI).

### Hospital outcome and six months follow-up

All patients demonstrated clinical stability, with no reports of chest pain. Cardiac enzyme levels were assessed, and antiplatelet and antithrombotic therapies were prescribed according to protocol. Throughout the six-month follow-up period, there were no recorded instances of lesion failure. However, one patient experienced a stroke, resulting in mortality (68 days after discharge). Importantly, no major adverse cardiovascular events (MACE) were observed within the cohort, as outlined in Table 4.

**Table 4:** Discharge and six months clinical outcomes.

Characteristics	Total (*n* = 31)	Percentage (%)
Antiplatelet therapy at discharge		
Acetylic salicylic acid (aspirin)	100%	
Clopidogrel	80%	
Prasugrel	10%	
Ticagrelor	10%	
Six months clinical outcomes		
Target lesion failure	0	0
Target vessel failure	0	0
Mortality	1	3.2
Cardiac	0	0
Non cardiac	1	3.2
Stroke	1	3.2
Myocardial infarction	0	0
Target-vessel	0	0
Non-target-vessel	0	0

## Discussion

During the initial implementation phase of R-PCI (April to September 2023), 31 cases involving a total of 37 lesions were treated with stenting, with no reported clinical complications attributable to robotic assistance. The clinical success rate was 97.3%, while the robotic success rate reached 94.6%. These favorable outcomes marked a notable advancement in the application of R-PCI, despite two instances requiring conversion to manual intervention.

The success rates observed in both clinical and robotic aspects align with findings reported in previous research studies, such as the Precision GRX study, where clinical success and robotic success were reported at 97.8% and 89.2%, respectively (14). Conversely, the CORA-PCI study demonstrated clinical success at 99.1% and robotic success at 91.7% (2, 14). These consistently high rates of clinical and robotic success underscore the feasibility of implementing R-PCI for routine clinical practice, emphasizing its safety and efficacy.

Furthermore, the results of the first R-PCI conducted in Japan in 2020 by Kotaro Kagiyama yielded compelling insights when comparing R-PCI utilizing the CorPath GRX robot to manual methods. The clinical success rate associated with R-PCI was found to be comparable to manual intervention, with rates of 93.3% and 94.6%, respectively, yielding a *p*-value of 0.97 (2, 14).

Notably, only one case (2.7%) exhibited complications (type B2/C) related to myocardial infarction due to occlusion of a small side branch, resulting in a TIMI II flow. In comparison, Pedro A. Lemos and Fabian J. Brunner reported incidence rates of 1.2% and 5.6%, respectively (6, 15). Such peri-procedural MIs stemming from SB occlusion can occur during any coronary intervention, particularly in arteries featuring severely narrowed SBs and calcification. The occlusion typically arises when dilating and intervening on the main branch, inadvertently blocking the SB. Crucially, this specific case involved a type B2/C lesion, highlighting that complications are associated with the inherent complexity of the lesion itself, rather than the robotic approach. The patient did not experience chest pain due to the small size of the occluded SB. Subsequently, the patient received medical management, and an echocardiogram revealed no adverse effects on myocardial contractility or other complications.

Two cases necessitated conversion to manual intervention due to the complexity of type C lesions. Firstly, in a LAD artery with a type C lesion characterized by calcification and diffuse stenosis, manual stenting became necessary after the robotic delivery of the stent failed. A Guidezilla catheter (Boston Scientific, Marlborough, MA, USA) was employed to support the PCI, resulting in TIMI 3 flow without any complications. Secondly, in a RCA with an abnormal origin from the anterior aspect of the right coronary sinus and a diffuse type C lesion, the catheter and wire placed robotically became dislodged during balloon delivery. Manual intervention involving the successful placement of two drug-eluting stents was subsequently performed.

The success rate of R-PCI in our study was notably high for the type A/B1 lesion group, achieving a 100% success rate. Particularly for type B2/C lesions characterized by their length, calcification, and complexity, requiring additional support from assistive devices, the intervention success rate reached 85.7%. In a study by E. Mahmud with 315 patients, 108 cases underwent R-PCI, with 78.3% of cases presenting type B2/C lesions. The study reported a conversion rate to manual intervention of 11.1% (2). This high success rate is partially attributed to the low volume of R-PCI procedures analyzed (*n* = 31). As this was the first clinical experience with the robotic system in Vietnam, the procedure was executed with meticulous caution and strict adherence to a learning protocol.

Furthermore, we acknowledge that a significant proportion of the lesions treated in our series were less complex (Type A/B1, 62.2%). This reflects our initial strategy: under the supervision of a manufacturer proctor, we deliberately selected less complex lesions to prioritize patient safety and facilitate a stable learning curve. Lesion complexity was then gradually increased as the interventionist gained technical proficiency with the robotic console. This cautious selection explains why Partial Manual Support was not required for the manipulation of coronary wires, balloons, or stents, unlike the findings in more complex cohorts. The only manual assistance necessitated was for the advancement of the IVUS catheter (*n* = 14), due to current hardware incompatibility. In comparison, Bay et al. reported a higher manual assistance rate of 19.9% (including 7.8% for Type A/B1 lesions), which often involved complex maneuvers such as bifurcation kissing or overcoming friction in long lesions scenarios that were less frequent in our early-stage experience (13).

During the 6-month follow-up period, no MACEs related to R-PCI were recorded. There was only one case of stroke and subsequent death, which was determined to be unrelated to the procedure. The recent one-year clinical outcome of R-PCI, such as those from the CORA-PCI registry reported a MACE rate of 7.8% and research conducted in Switzerland a rate of 5% (5–7). Alongside the consistently high success rates of R-PCI observed worldwide, the results from our study once again underscore its safety, efficacy, and feasibility in clinical practice.

### Lessons to learn during the robotic-PCI implementation process

The Corindus robotic system, integrated with technIQ technology features such as Spin, RoR, Wiggle, dotter, and constant speed, has significantly enhanced the proficiency in navigating guidewires through intricately compromised coronary arteries. The system's exceptional capability to manipulate and stabilize the guidewire during interventions mitigates the risk of complications associated with inadvertent loss of control over the guidewire. The spin technique involves rotating the wire and is particularly effective for routine navigation through vessels with larger diameters and complex curves. In contrast, the wiggle technique employs rapid, alternating clockwise and counterclockwise rotations of the wire during advancement, making it advantageous for navigating tight or narrow segments within smaller vessels. Additionally, the “rotate on retract” method automatically applies a 270-degree rotation to the wire as it is withdrawn. This feature is especially beneficial when encountering multiple side branches, as it facilitates redirection of the wire toward the main vessel if it inadvertently enters a side branch.

Collectively, these techniques replicate the wiring practices employed by experienced interventional cardiologists, providing a valuable training platform that reduces the learning curve for new cardiologists. As Yong et al. noted in a previous study, these methods significantly enhance procedural stability and efficiency, offering substantial support to interventional cardiologists during interventions (16).

The integration of pre-programmed leads and control buttons on the robotic console effectively mitigates the issue of hand tremors, which may affect some interventional cardiologists, particularly older practitioners. Additionally, these advanced robotic systems are impervious to external influences such as psychological stress, environmental conditions, operator experience, and age (16). By providing enhanced stability during interventions, robotic systems contribute significantly to procedural precision.

Notably, the objectives of robotic-assisted percutaneous coronary interventions (R-PCI), as well as other surgical robotic systems, extend beyond stability to include minimizing complications arising from human factors, such as manual instability. Drawing from the demonstrated success of robotic systems in achieving surgical precision across various disciplines, R-PCI represents a remarkable milestone in guidewire manipulation, substantially reducing the risk of complications associated with hand tremors (17).

The utilization of the Corindus robotic system for coronary artery intervention presents numerous advantages. By allowing the interventional cardiologist to remotely control the procedure from a comfortable control room, this system effectively reduces radiation exposure for both the physician and technical support staff. This transition in workflow occurs subsequent to sheath placement and robotic arm installation. Moreover, the system seamlessly integrates with the majority of existing interventional tools and exhibits a notably high success rate for type A, B1, and B2 coronary lesions, as detailed in Table 5.

**Table 5:** Classification of lesions.

Characteristics		Technical aspect	
		Successful	Failure
ACC/AHA coronary lesions type	Type A/B1	23 (100%)	0 (0%)
	Type B2/C	12 (85.7%)	2 (14.3%)
Calcification Degree	Severity	2 (50%)	2 (50%)
	Mild/moderate	14 (100%)	0 (0%)
	No calcium	19 (100%)	0 (0%)

American college of cardiology/American heart association (ACC/AHA).

### Personnel and the learning curve

The inclusion of two experienced interventionalists was deemed necessary for optimal patient safety and procedural efficiency during the adoption of the system. The assistant operator primarily facilitated tasks requiring manual intervention (such as managing challenges with support tools) and received direct proctoring and training from the Senior Operator, thereby minimizing the learning curve for new physicians.

When the center's experience and proficiency with the Corindus system increase, the number of required operators has reduced to a single primary operator for routine, less complex cases, allowing the full potential of personnel reduction to be realized, especially concerning non-essential personnel within the sterile field. In these routine procedures, the remaining assistant personnel only provide minimal manual assistance strictly limited to device installation onto the cassette. This evolution aligns with the findings of other centers that have demonstrated reduced essential staff requirements after the initial implementation period.

### Implementation challenges

However, there are certain limitations to consider. Despite its efficacy, several interventional support tools, including intravascular ultrasound (IVUS), optical coherence tomography (OCT), and extension catheters, can pose challenges during utilization and often necessitate manual assistance. When using IVUS in R-PCI, the IVUS catheter will be placed in the position of the coronary angioplasty balloon. During the procedure, we encountered challenges in advancing the guidewire using the robotic system. As a result, we had to manually push the guidewire with the assistance of a second interventional cardiologist present in the procedure room. Furthermore, we have not utilized OCT for coronary interventions performed with robotic systems and therefore lack experience with its application in this context. The lesions in these cases were relatively mild, and the use of OCT was not strongly indicated compared to IVUS. Current robotic systems are only partially compatible with intracoronary imaging devices, necessitating the selective and context-specific application of newer methods (7, 9, 14).

The introduction of R-PCI has exhibited encouraging indications, displaying commendable adaptability to diverse clinical scenarios (18). However, its implementation has encountered several challenges, notably in the realm of cost. Given its status as a novel and sophisticated technology, R-PCI is likely to entail substantial expenses, encompassing both initial acquisition and subsequent maintenance. Such elevated costs may render it financially prohibitive for the majority of healthcare facilities.

Moreover, the significant investment required for R-PCI can contribute to escalated procedure fees, potentially constraining patient accessibility. This financial barrier may impede many patients from availing themselves of procedures utilizing this advanced technology (19).

### Limitations

While this study offers valuable insights into the feasibility and success of robotic percutaneous coronary intervention (R-PCI), several limitations must be acknowledged. The small sample size of 31 cases restricts the statistical robustness of the findings, and the selective inclusion of patients with a preexisting understanding of their coronary anatomy may limit the applicability of these results to more complex or emergent scenarios. Furthermore, the study's single-center design, conducted by a specialized team within a familiar catheterization laboratory, likely contributed to the high success rates. This controlled setting does not account for variations in expertise, equipment, or workflows that may exist across other institutions, making the learning curve and procedural adjustments specific to this center.

We acknowledge a limitation regarding data granularity: highly detailed quantification (e.g., number of pull/push actions or device setup time) was not captured prospectively and is unreliable for retrospective reconstruction. Instead, we focused on providing clear quantitative limits in the methods and confirming the 100% setup requirement and the 14 cases requiring IVUS support in the results, collectively substantiating the minimal nature of the required assistance.

Additionally, the absence of long-term follow-up data precludes evaluation of R-PCI's sustained outcomes, such as stent durability and the recurrence of coronary events. To address these limitations, future multicenter studies involving larger and more diverse patient cohorts, alongside extended follow-up periods, are essential. Such investigations would help validate the findings, address procedural variability, and assess the long-term effectiveness and cost-effectiveness of R-PCI in broader clinical contexts.

Finally, a major limitation must be noted: the CorPath GRX robotic system utilized in this study was retracted from the market by Siemens Healthineers in the end of 2023. While this affects the immediate applicability of our specific findings, the discontinuation is understood to be a strategic step to facilitate the launch of a next-generation platform, confirming the manufacturer's continued commitment to robotic PCI.

## Data Availability

The raw data supporting the conclusions of this article will be made available by the authors, without undue reservation.
